# Lithium treatment and thyroid abnormalities

**DOI:** 10.1186/1745-0179-2-23

**Published:** 2006-09-12

**Authors:** Alberto Bocchetta, Andrea Loviselli

**Affiliations:** 1 Section on Clinical Pharmacology, "Bernard B. Brodie" Department of Neurosciences, University of Cagliari, via Ospedale 46, 09124 Cagliari, Italy; 2 "Mario Aresu" Department of Internal Medical Sciences, Policlinico Universitario, University of Cagliari, S.P. Monserrato-Sestu, 09042 Monserrato, Cagliari, Italy

## Abstract

**Background:**

Although the interactions between lithium treatment and thyroid function have long been recognised, their clinical relevance is still controversial. This paper sets out a review of the literature to date, considering that lithium still represents the gold standard among prophylactic treatments of manic-depression several decades after its introduction.

**Method:**

PubMed database was used to search for English-language articles relating to lithium treatment and thyroid function. As the amount of relevant papers totalled several hundreds, this review refers to previous reviews, especially with regard to older literature. Moreover, the authors particularly refer to a series of studies of thyroid function performed in a cohort of patients at different stages of lithium treatment, who were followed up by their group from 1989 onwards.

**Results:**

The main findings from this review included: a) lithium definitely affects thyroid function as repeatedly shown by studies on cell cultures, experimental animals, volunteers, and patients; b) inhibition of thyroid hormone release is the critical mechanism in the development of hypothyroidism, goitre, and, perhaps, changes in the texture of the gland which are detected by ultrasonic scanning; c) compensatory mechanisms operate and prevent the development of hypothyroidism in the majority of patients; d) when additional risk factors are present, either environmental (such as iodine deficiency) or intrinsic (immunogenetic background), compensatory potential may be reduced and clinically relevant consequences may derive; e) hypothyroidism may develop in particular during the first years of lithium treatment, in middle-aged women, and in the presence of thyroid autoimmunity; f) thyroid autoimmunity is found in excess among patients suffering from affective disorders, irrespective of lithium exposure; g) in patients who have been on lithium for several years, the outcome of hypothyroidism, goitre, and thyroid autoimmunity do not much differ from those observed in the general population; h) hyperthyroidism and thyroid cancer are observed rarely during lithium treatment.

**Recommendations:**

Thyroid function tests (TSH, free thyroid hormones, specific antibodies, and ultrasonic scanning) should be performed prior to starting lithium prophylaxis. A similar panel should be repeated at one year. Thereafter, annual measurements of TSH may be sufficient to prevent overt hypothyroidism. In the presence of raised TSH or thyroid autoimmunity, shorter intervals between assessments are advisable (4–6 months). Measurement of antibodies and ultrasonic scanning may be repeated at 2-to-3-year intervals. The patient must be referred to the endocrinologist if TSH concentrations are repeatedly abnormal, and/or goitre or nodules are detected. Thyroid function abnormalities should not constitute an outright contraindication to lithium treatment, and lithium should not be stopped if a patient develops thyroid abnormalities. Decisions should be made taking into account the evidence that lithium treatment is perhaps the only efficient means of reducing the excessive mortality which is otherwise associated with affective disorders.

## Background

Abnormalities in thyroid function have concerned clinicians and patients since the introduction of lithium in the treatment of manic-depression. Goitre was one of the first described potential side effects of this treatment [1] and prompted subsequent studies on the interactions between lithium and thyroid function [2]. Moreover, it soon became evident that lithium treatment is at times associated with clinical hypothyroidism [3]. Decades of clinical use of lithium and availability of new diagnostic tools for thyroid abnormalities have extended the interest to other aspects, including autoimmunity, hyperthyroidism, and morphological changes.

The advent of alternative treatments for recurrent affective disorders warrants a review of the clinical relevance of lithium-related side effects, including those regarding thyroid function. The amount of scientific papers dealing with lithium and thyroid function totals several hundred; therefore we shall refer to previous reviews, especially with regard to older literature [4,5]. We will also refer to a series of studies of thyroid function performed in a cohort of 150 patients at different stages of lithium treatment, who were followed up by our group from 1989 onwards [6-9].

The following important points will also be addressed: a) the emerging evidence that thyroid abnormalities, in particular autoimmunity, are found in excess among patients suffering from affective disorders, irrespective of lithium exposure [10,11]; b) the evidence that lithium treatment, despite its potential toxicity and side effects, is perhaps the only drug capable of reducing the excessive mortality which is otherwise associated with affective disorders [12,13].

## Goitre and ultrasonic scan abnormalities

The goitrogenic effect of lithium was observed early after its introduction in the treatment of manic-depression. In 1968, Schou and coworkers reported a prevalence of goitre of 3.6% and calculated an annual incidence of 4% among patients on continuous lithium compared with a 1% incidence in a geographically matched general population [1]. Nowadays, despite such a long history, controversy still persists regarding the relevance of lithium-induced goitre. Large long-term prospective studies using reliable methods of detection (i.e. ultrasonic scans) are lacking, and a wide range of prevalence rates (0–60%) is reported from existing cross-sectional studies (for review see [4]). Differences in the methods of detection, together with variation in the geographical origin of patients (especially related to iodine intake), are perhaps the main reason for such discrepancies. The most reliable existing study of goitre in manic-depressive patients, though cross-sectional, is perhaps that by Perrild et al [14]. Ultrasonically determined thyroid size was larger than expected (according to age and weight of subjects) among patients treated with lithium for 1–5 years (44%) or more than 10 years (50%) than in patients who had never received lithium (16%).

As the relevance of goitre among lithium-treated patients is still debated, the attitude of clinicians varies: the main question is whether or not TSH-suppressive treatment is to be started in lithium patients as soon as goitre is detected even in the absence of hypothyroidism [15,16]. Some authors even recommend prophylaxis with levothyroxine in all patients starting lithium if they come from goitre-endemic areas [17]. Their suggestion was based on an 87% prevalence found in lithium patients from a region where the general prevalence ranged from 20% to 30%. On the other hand, Lazarus [15] does not argue in favour of treatment, also based on the possible occurrence of lithium-associated thyrotoxicosis (see the specific section). There are other reasons for caution in the use of TSH-suppressive levothyroxine. Besides the general limitations of TSH-suppressive treatment including advanced age, cardiovascular problems, and osteoporosis, one additional problem in lithium patients may regard their acceptance of chronic polypharmacy. Unpublished data from our cohort revealed that, after at least 10 years of lithium treatment, up to two-thirds of patients were taking chronic psychotropic medication in addition to lithium and 31% were taking chronic medication for other medical conditions apart from levothyroxine (including 18% on antihypertensive medication).

Another relevant observation from our cohort is that the rate of patients with a visible and/or palpable goitre (which attained 51% when first examined [6]) decreased at follow up [8,9], even in the absence of levothyroxine treatment and despite continuous exposure to lithium. This is similar to the results from the Whickham Survey of the general population [18], and implies that the severity of goitre and the presence of additional factors should be taken into account in the decision of whether or not to prescribe treatment with TSH-suppressive levothyroxine. The latter, moreover, would not affect one of the mechanisms underlying lithium-induced increase in thyroid volume, i.e. its direct effect on the growth of thyroid cells, as shown *in vitro *[19].

With regard to ultrasonic scan abnormalities apart from increased volume, in our cohort with at least six-year lithium treatment, up to 97% of women and 69% of men without evidence of thyroid circulating antibodies showed abnormalities (reduced echogenicity, non-homogeneous echopattern, and/or presence of nodules) [8]. Ultrasonic scan abnormalities may indeed be associated to the inhibition of thyroid hormone release, the main effect exerted by lithium on thyroid function [2]. A more recent German ultrasound study reported different results, as thyroid echogenicity was found to be similar between 20 patients who had been on lithium for at least 6 months and 20 age- and gender-matched controls [20]. We have addressed elsewhere the potential value of thyroid echography in the identification of patients at risk of developing autoimmune hypothyroidism during long-term lithium therapy [21]. The relevance of thyroid nodules will be discussed in the section on tumours.

## Hypothyroidism

Hypothyroidism, irrespective of association with goitre, has been one of the main concerns regarding lithium treatment since the early 1970s [3]. Several interactions between lithium and thyroid function have been evidenced (for review see [4]). Lithium is concentrated by the thyroid and inhibits thyroidal iodine uptake. It also inhibits iodotyrosine coupling, alters thyroglobulin structure, and inhibits thyroid hormone secretion. The latter effect is critical to the development of hypothyroidism and goitre. Many studies have investigated prevalence rates of hypothyroidism. As already mentioned with regard to goitre, differences in criteria (for example overt versus subclinical hypothyroidism) and study population (gender ratio, geographical origin of patients, iodine intake, proportion of subjects with autoimmunity, etc) may explain the wide range of prevalence rates (0–23%) from existing cross-sectional studies (for review see [4,5]). In a retrospective study of 718 lithium patients, Johnston and Eagles [22] found a 10.4% prevalence of clinical hypothyroidism, calling for an upward revision of generally quoted rates of '90s reviews (2–5%). They identified a higher risk in women, especially those starting lithium in middle age (hypothyroidism prevalence > 20%). They calculated retrospectively annual incidence figures of 2.17% in women and 0.68% in men, which are substantially higher than the community incidences of hypothyroidism reported by Vanderpump *et al *(0.41% and 0.06%) [18].

Kirov *et al *[23] have recently published a study of thyroid disorders in 274 lithium patients, including 57 who had been observed longitudinally for between 1 and 7 years. The risk for hypothyroidism was especially increased in women over the age of 50. Of the 33 women followed up prospectively, 4 developed hypothyroidism, corresponding to an incidence of 2.74%. The latter figure is a nearly eight-fold increase over the mean incidence of 0.35% among middle-aged women in the Whickham Survey [18].

In our prospective evaluation [9], incidence of cases requiring replacement treatment with levothyroxine (2.1% in women and 0.3% in men) was very similar to the incidence of hypothyroidism reported by the UK studies [22,23]. In the absence of a control group, incidence may be compared with published data from the general population, but there may be wide variation due to differences in geographical areas and in criteria for hypothyroidism. Overt hypothyroidism is becoming rare as replacement treatment is now prescribed in the presence of subclinical hypothyroidism (raised TSH plus reduced thyroxine concentrations). Current epidemiological studies, including the follow up of the Whickham Survey [18], usually focus on intention-to-treat. On the other hand, lithium patients often receive replacement treatment with levothyroxine in the presence of repeated raised TSH concentration alone, which may lead to an overestimation of incidence when compared with community data. In our 10-year follow-up study [9], we compared rates with the subset of data from the Whickham Survey published by Tunbridge *et al *[24], who included figures of raised TSH concentrations (above 6 μU/mL) alone. Thus, our conclusion that there is a similarity between rates of hypothyroidism in lithium patients and those in the general population [9], diverged from the conclusions of the UK studies [22,23]. Perhaps, the truth is somewhere between the two extremes. In any case, whatever the proportion of cases of hypothyroidism that can be attributed to lithium treatment, we confirmed the relevance of other risk factors, such as gender and presence of thyroid autoimmunity. The hierarchy of risk between our lithium-cohort and the community is similar, as the highest annual rates of hypothyroidism are observed in antibody-positive subjects, followed by antibody-negative women, whereas risk is apparently null in antibody-negative men from both settings.

## Hyperthyroidism

Cases of hyperthyroidism have been associated with lithium treatment since the 1970s [25], but less commonly compared with hypothyroidism and goitre. Indeed, being the main effect of lithium a reduction of thyroid hormones, it has even been used in the treatment of thyrotoxicosis [26]. There are conflicting opinions regarding the relevance of hyperthyroidism during lithium treatment. Sirota *et al *[27] reported nine cases although, based on the relatively small number of case reports in the literature and the diffusion of lithium treatment, they concluded that "lithium therapy does not cause hyperthyroidism". Conversely, Barclay *et al *[28] reported 14 cases and calculated retrospectively a higher than expected incidence of hyperthyroidism. Of the 33 women followed up prospectively by Kirov *et al *[23], one developed hyperthyroidism over 146 patient-years. In our cohort, one case of hyperthyroidism was observed in a woman at the last follow-up (to be published), when the observation period for women totalled 680 patient-years, corresponding to an annual rate of 0.1%. The latter rate does not diverge from the incidence reported for the female general population by the Whickham Survey [18].

## Antithyroid antibodies

Lithium affects many aspects of cellular and humoral immunity *in vitro *and *in vivo*, but it is controversial whether lithium *per se *can induce thyroid autoimmunity. Prevalence of specific thyroid antibodies among lithium-treated patients varies across studies. It is however important to underline once again the effects of age and gender. Women are known to express thyroid autoimmunity more frequently than men. This tendency is more obvious in the middle age range. Initial and final prevalence rates from our lithium cohort (women, 21 to 27%; men, 4 to 10%) [9] were within the ranges observed in similar age and gender subgroups from the Whickham Survey [18].

Several prospective studies, though reporting fluctuations in antibody titres, failed to detect differences between pre- and post-lithium prevalence rates of autoimmunity [29-31]. However, an intriguing observation from our cohort was the development of thyroid antibodies in young subjects of both sexes within a few years of lithium exposure [7]. Nevertheless, annual incidence rates in our patients after several years of lithium treatment (1.3–1.5%) did not much differ from the ranges reported for the general population, with maximum values of approximately 2% in women aged over 45 [18,24].

It is noteworthy, as mentioned above, that thyroid autoimmunity has been found associated with affective disorders, irrespective of treatment [11,32]. Kupka *et al *[11] found thyroperoxidase antibodies in 64 of 226 (28%) bipolar outpatients. In the latter, autoimmunity was associated with thyroid failure, but not with age, gender, mood state, rapid cycling, or lithium exposure. Indeed, 12 of 35 (34.3%) who had never received lithium had thyroperoxidase antibodies.

## Tumours

Lithium treatment has not been associated with thyroid tumours, apart from nodular goitre. In our cohort with at least 6 years of lithium treatment, ultrasound revealed nodules in 47% of women and 24% of men [8]. Similar prevalence rates have been found in the Sardinian general population (to be published). Multinodular goitre is particularly prevalent in iodine-deficiency areas, whereas female gender and advancing age represent additional risk factors. The major concern regards the potential malignant nature of nodules, which requires further diagnostic procedures, such as fine needle aspiration with cytology. The latter revealed malignancy (papillary carcinoma) in a woman from our cohort at the last follow-up (to be published). No other cases were evidenced and the annual incidence rate in women (over 680 patient-years) was 0.1%.

## Conclusion

The clinical relevance of thyroid dysfunction during long-term lithium is an important issue, considering that lithium still represents the gold standard among prophylactic treatments of manic-depression several decades after its introduction. Lithium definitely affects thyroid function as repeatedly shown by studies on cell cultures, experimental animals, volunteers, and patients. Inhibition of thyroid hormone release is the critical mechanism in the development of hypothyroidism, goitre, and, perhaps, changes in the texture of the gland which are detected by ultrasonic scanning. Compensatory mechanisms operate and prevent the development of hypothyroidism in the majority of patients. When additional risk factors are present, either environmental (such as iodine deficiency, dietary goitrogens, cigarette smoking) or intrinsic (immunogenetic background), compensatory potential may be reduced and clinically relevant consequences may derive. Hypothyroidism may occur in particular in middle aged-women and/or in the presence of thyroid autoimmunity. Hyperthyroidism and thyroid cancer are less common.

On clinical grounds, the following procedures are recommended in lithium patients. Assessment of thyroid function prior to starting lithium prophylaxis should include measurement of serum concentrations of TSH, FT3, FT4, AbTPO, and ultrasonic scanning. A similar panel should be repeated at one year. Thereafter, annual measurements of TSH may be sufficient to prevent overt hypothyroidism. In the presence of subclinical hypothyroidism (raised TSH), shorter intervals between assessments are advisable (4–6 months). Measurement of AbTPO and ultrasonic scanning may be repeated at 2-to-3-year intervals. The patient must be referred to the endocrinologist if TSH concentrations are repeatedly abnormal, and/or goitre or nodules are detected.

Finally, in our opinion, in view of the availability of adequate therapeutic means, the presence of thyroid function abnormalities should not constitute an outright contraindication to lithium treatment, contrary to arguments often put forward and practiced to date. Similarly, lithium should not be stopped if a patient develops thyroid abnormalities. Any decision should be made taking into account the evidence that lithium treatment, despite its potential toxicity and side effects, is perhaps the only efficient means of reducing the excessive mortality which is otherwise associated with affective disorders.
